# Peritoneal adhesion: it can be life-threatening, and life-saving

**DOI:** 10.1186/1471-2369-13-113

**Published:** 2012-09-19

**Authors:** Jiun-Chi Huang, Szu-Chia Chen, Tsung-Kun Yang, Fang-Jung Yu, Fu Ou-Yang, Jer-Ming Chang

**Affiliations:** 1 Department of Internal Medicine, Kaohsiung Municipal Hsiao-Kang Hospital, 482 San-Ming Rd, Hsiao-Kang District, Kaohsiung, 812, Taiwan; 2 Division of Nephrology, Kaohsiung Medical University Hospital, 482 San-Ming Rd, Hsiao-Kang District, Kaohsiung, 812, Taiwan; 3 Division of Gastroenterology, Kaohsiung Medical University Hospital, 482 San-Ming Rd, Hsiao-Kang District, Kaohsiung, 812, Taiwan; 4 Department of Surgery, Kaohsiung Medical University Hospital, 482 San-Ming Rd, Hsiao-Kang District, Kaohsiung, 812, Taiwan; 5 Faculty of Renal Care, School of Medicine, Kaohsiung Medical University, Kaohsiung, Taiwan

**Keywords:** Peritoneal dialysis, Peritonitis, Ultrafiltration failure, Peritoneal adhesion, Encapsulating peritoneal sclerosis

## Abstract

**Background:**

The inevitable post-inflammatory fibrosis and adhesion often compromises future treatment in peritoneal dialysis patients. Here, we describe a patient who experienced an unusual form of peritoneal adhesion that made her give up peritoneal dialysis. However, its unique pattern also saved her from infection caused by bowel perforation.

**Case presentation:**

The female patient discontinued peritoneal dialysis due to gradual dialysis inadequacy. Two months after shifting to hemodialysis with generally improved sense of well-being and no sign of abdominal illness, she was admitted to remove the Tenckhoff catheter. The procedure was smooth, but fever and abdominal pain not at the site of operation developed the next day. Abdominal ultrasound showed the presence of ascites and aspiration revealed slimy, green-yellowish pus that gave a negative result on bacterial culture. Abdominal computed tomography (CT) with oral contrast medium was performed, but failed to demonstrate the suspected bowel perforation. The examination, however, did show accumulation of pus inside the abdomen but outside the peritoneal cavity. We drained the pus with two 14-F Pig-tail catheters and the total amount of drainage approached 4000 ml. The second CT was performed with double dose of the contrast medium and found a leak of the contrast from the jejunum. She then received laparotomy and had the perforation site closed.

**Conclusions:**

In summary, this uremic patient suffered from pus accumulation inside her abdomen without obvious systemic toxic effect. The bowel perforation and pus formation might be caused by repeated peritonitis, but the peritoneal adhesion itself might also isolate her peritoneal cavity from the anticipated toxic injuries of bowel perforation.

## Background

Peritoneal dialysis (PD) is a modality chosen by approximately 11% of end-stage renal disease (ESRD) patients in 2004 and 15.8% in 2009 globally [1]. The primary advantage of PD is patients’ ability to undertake treatment without having to visit a medical facility frequently. It has comparable risks attributed to the permanent catheter that may introduce bacteria into the abdomen and cause peritonitis, the most common acute complication of PD [2]. PD-related peritonitis is largely caused by cutaneous Gram’s (+) bacteria, although Gram’s (−) bacteria peritonitis has become more and more common, and can usually be treated effectively by appropriate antibiotics [3]. However, the inevitable post-inflammatory fibrosis and adhesion often compromise patients’ future treatment. Manifestations of impaired peritoneal function include ultrafiltration failure, PD inadequacy, abdominal symptoms of bowel obstruction, and malnutrition, thus threatening patients’ lives [4]. The most severe form estimated to occur in roughly 2.5% of patients is encapsulating peritoneal sclerosis (EPS), in which the bowels become obstructed due to the growth of a thick layer of collagen within the peritoneum [5]. Mortality rate of EPS can be as high as 50%. The management of peritoneal fibrosis and adhesion itself is usually not effective enough to restore the peritoneal function to its original level. Therefore, peritonitis rate is often employed to audit the quality assurance in PD practice and it also reflects faithfully whether the education offered to the patients and prevention measures are successful [6].

Here, we describe a patient who experienced an unusual form of EPS that made her give up PD. However, its unique pattern also saved her from infection caused by bowel perforation.

## Case presentation

**Figure 1:**
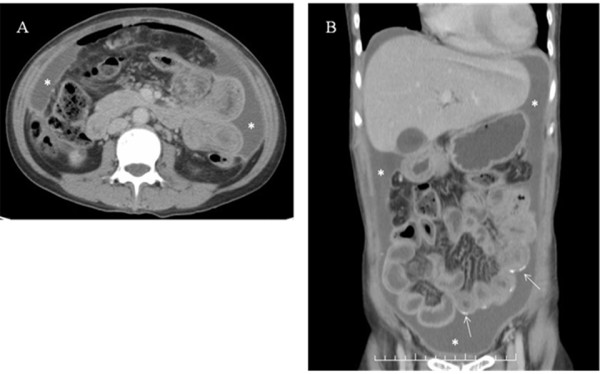
A and B. Large amount of pus (*). The pus was located inside the abdomen but did not involve the peritoneal cavity. Several peritoneal calcifications were seen (arrow), most likely due to the sequel of previous peritonitis.

**Figure 2:**
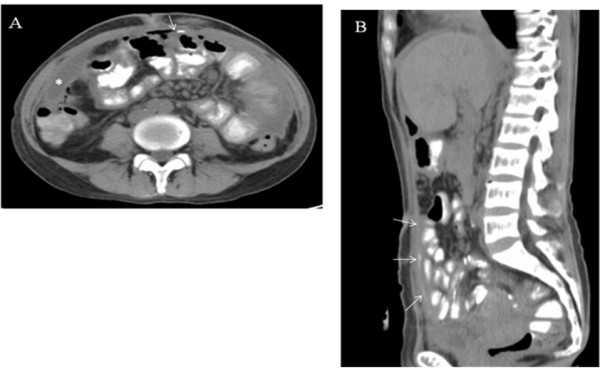
**A and B. Repeated CT scan with patient ingesting 200 ml of Ultravist® medium.** Both transverse **(A)** and sagittal **(B)** sections showed suspicious medium leak (arrow) but the amount was small and inconclusive to reveal the site of leakage. The pus (*) had been partially drained.

**Figure 3:**
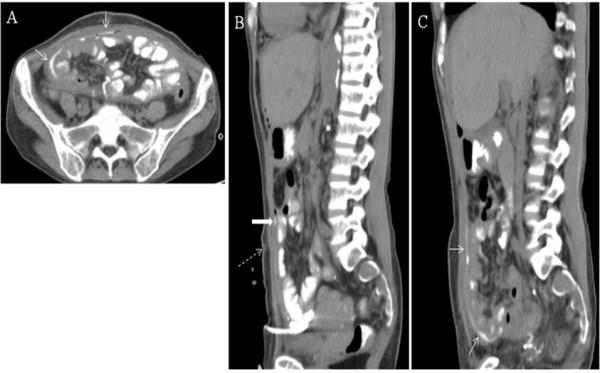
**A, B and C. Peated CT scan when pus was completely drained and patient ingested 400 ml of Ultravist® medium.** The transverse image suggested a cocoon-like conformation which kept the leaking medium from entering the peritoneal cavity (arrow in Figure 3A and 3C). Figure 3B shows the favored site of perforation (solid arrow) near the original exit site of PD catheter (dotted arrow). It was later proved by the laparotomy finding.

The female patient has been followed up for chronic glomerulonephritis for several years before she finally decided to take continuous ambulatory PD (CAPD) as her renal replacement therapy. Her CAPD continued uneventfully for more than seven years until she had the first peritonitis caused by *Staphylococcus aureus*. It was successfully treated with intraperitoneal cephalosporin antibiotics for ten days and left no significant clinical complication. The second peritonitis was noted in more than one year after the first. *Pseudomonas aeruginosa* was cultured from the dialysate and her abdominal symptoms were severe. She did not agree to the suggestion to remove the catheter and instead, was treated with both intraperitoneal and intravenous ceftazidime. The treatment course lasted for almost one month, but eventually her abdominal symptoms gradually improved and the dialysate became clear. She went on caring for herself with CAPD in the following year. However, drain of dialysate gradually became inadequate and peripheral edema developed. Her body weight was not increased (39–40 Kg) despite of the presence of marked edema. Ultrafiltration failure caused by peritoneal adhesion was diagnosed and she finally consented to giving up CAPD and shifting to hemodialysis (HD), three times a week and 3.5 hours per session. Drainage fluid of PD solutions had been clear in every exchange till the termination of PD treatment. HD went on smoothly and removed all the accumulated edema until her body weight became 33 Kg. She regained her appetite and her body weight gradually increased to 36–37 Kg in the following six weeks. General sense of well-being improved significantly and she was able to go abroad twice for three-day tours. She was then admitted to remove the PD Tenckhoff catheter after maintaining stable HD for two months. The operation was successful but unfortunately, fever up to 38.7°C was noted the next day. Blood leukocyte count was 10780 per ml and C-reactive protein was 129 mg/dl. Oral acetaminophen and intravenous broad-spectrum antibiotics were given under the suspicion of procedure-related infection and blood was sent to the laboratory for culture which gave negative result. Fever subsided after two days, but she started to complain of abdominal pain and tenderness, not at the site of operation. Abdominal ultrasound was performed and showed large amount of ascites. Bedside paracentesis with 19-F syringe revealed slimy green-yellowish pus which was immediately sent to the laboratory for microbial analysis. Surprisingly, the microscopic and culture examination reported negative for any bacteria. For detailed anatomic analysis and further management, she received abdominal computed tomography (CT) examination which suggested the formation of a cocoon wrapping the intestines. Large amount of ascites (with much higher CT number than water) was shown outside the cocoon (Figure 1A and 1B), and two 14-F pigtail catheters were placed to drain the pus. The total amount of drainage (by two catheters) in the following seven days approached 4000 ml. Repeated CT examination while the patient ingesting 200 ml Ultravist® contrast medium was performed to locate the suspicious bowel perforation, but the result was inconclusive (Figure 2A and 2B). We discontinued the antibiotic treatment and removed the catheters after a near-complete drainage and arranged another CT scan with the patient ingesting 400 ml contrast medium. The ingested medium was found to leak from the jejunum and spread under the abdominal wall as a thin layer (Figure 3A-C), but not inside the peritoneal cavity. She then received a laparotomy and extensive adhesion of the visceral peritoneal membrane that wrapped all the intestines into a cocoon-like pattern was seen during operation, along with a small amount of residual ascites outside the cocoon. The surgeon successfully located a small perforation that matched the suspected leakage site and closed it directly. She recovered quickly from the procedure with no sign of infection. Regular maintenance HD was given and both her appetite and nutritional status were improved.

Six months after surgery, her body weight increased gradually to 37–38 Kg. There was no edema, and abdominal symptoms were absent.

## Conclusions

In this article, we described an unusual clinical course of a CAPD patient who suffered from peritoneal adhesion due to CAPD-related peritonitis. The complication was serious enough to compromise her CAPD, but was so specific that it prevents the invasion of a potentially lethal infection.

Peritonitis remains a leading complication of PD and contributes greatly to morbidity and mortality of PD patients [7]. Severe peritonitis can cause peritoneal membrane failure and stays as the major cause of discontinuing PD and shifting to HD. Peritonitis infected by Gram’s (−) bacteria has become more common, although Gram’s (+) organisms remain the major causes [3]. Previous study has shown that *Pseudomonas aeruginosa* peritonitis is generally severe and associated with longer duration of admission, more severe peritoneal membrane damage, and higher rate of catheter removal and transfer to HD [8]. Therefore, some even suggested prompt catheter removal upon diagnosis of *Pseudomonas* peritonitis for better outcomes [9]. Our patient, in her second peritonitis event, was successfully treated with antibiotics for one month and continued her PD treatment. However, PD inadequacy soon became evident and this was also proved by follow-up peritoneum equilibration test. Her Kt/V decreased from 1.99 to 1.44, and weekly creatinine clearance dropped from 56 liters per week to 41 liters per week. These findings and severe edema persuaded her to switch to HD.

We had cautiously considered the possible diagnosis of encapsulated peritoneal sclerosis (EPS), the most serious complication of long-term PD therapy, because the treatment of EPS was usually unsatisfactory and outcome could be poor with high mortality rate [10]. This patient presented several risk factors associated with EPS development, including long duration of PD, young age, exclusive use of dextrose solution, peritonitis, and late ultrafiltration failure. She also suffered from gradual reduction of appetite, progressive weight loss (obvious peripheral edema but unvarying body weight), malnutrition (serum albumin < 3.5 g/dl for almost a year before shifting to HD), and favorable structural finding noted during laparotomy. However, she did not experience symptoms and signs of bowel obstruction that are common in patients with severe EPS, such as progressive abdominal pain, abdominal distention, or difficulties in defecation. The poor appetite and peripheral edema were effectively corrected by changing treatment modality without specific treatment for EPS, such as enterolysis [11], tamoxifen [12], or immunosuppressive agents [13]. In the most severe form of EPS, a sclerotic layer may completely cover the intestines, which gives them the appearance of a cocoon. The CT images supported the diagnosis of EPS and cocoon formation, but they also showed that the adhesion and fibrosis were relatively mild in the peritoneum between intestines. This might partly explain the absence of excruciating abdominal symptoms of bowel obstruction.

This patient did not experience any traumatic event that might lead to her bowel perforation. The most reasonable explanation would still be the adhesion between the intestines and abdominal wall at the insertion site. She had kept her Tenckhoff catheter for about two months before removal. With hindsight, the catheter might have been pulled unintentionally, thus tearing and bowel wall. Nevertheless, the tear was too small to cause significant abdominal symptoms, as shown by the clinical wellness and improvement in the period before Tenckhoff catheter removal. The leakage still was manifested by large amount of intra-abdominal pus accumulating gradually between the parietal and visceral peritoneal membranes, within the same period of time. It’s very likely that the peritoneal cavity was completely covered by a fibrotic layer and the pus did not gain access into the intestinal loops. Therefore, this patient could live a life of wellness devoid of any significant gastrointestinal symptoms in those two months of HD. Pus was later drained smoothly with pigtail catheters and the bowel perforation was then successfully closed.

In summary, this patient had suffered from peritonitis-related peritoneal adhesion severe enough to cause ultrafiltration failure that made her change dialysis modality. Diagnosis of EPS was supported by image findings. Nevertheless, the pattern of adhesion was so specific that kept the pus out of her intestinal loops and salvaged her from the anticipated abdominal catastrophe.

## Consent

Written consent was obtained from the patient’s family for the publication of the case report and its accompanying images.

## Competing interest

The authors declared that they have no conflicting interests.

## Authors’ contributions

YFJ, YFO and CJM treated the patient, CSC and YTK collected data and drafted the manuscript together with CJM. All authors read and approved the final manuscript.

## Pre-publication history

The pre-publication history for this paper can be accessed here:

http://www.biomedcentral.com/1471-2369/13/113/prepub
